# Changes in Stress-Strain Index and Corneal Biomechanics in Granular Corneal Dystrophy

**DOI:** 10.3390/jcm11216571

**Published:** 2022-11-05

**Authors:** Hamidreza Heidari, Hamed Momeni-Moghaddam, Khosrow Jadidi, Shiva Pirhadi, Majid Moshirfar

**Affiliations:** 1 Rehabilitation Research Center, Iran University of Medical Sciences, Tehran 14496-14535, Iran; 2 Rehabilitation Sciences Research Center, Zahedan University of Medical Sciences, Zahedan 43463-98167, Iran; 3 Vision Health Research Center, Semnan University of Medical Sciences, Semnan 35147-99442, Iran; 4 Department of Biomedical Engineering, Science and Research Branch, Islamic Azad University, Tehran 14496-14535, Iran; 5 Hoopes Vision Research Center, Hoopes Vision, 11820 S. State St. #200, Draper, UT 84020, USA; 6 John A. Moran Eye Center, University of Utah School of Medicine, Salt Lake City, UT 84132, USA; 7 Utah Lions Eye Bank, Murray, UT 84107, USA

**Keywords:** cornea, corneal biomechanics, Corvis ST, granular corneal dystrophy

## Abstract

Background: The aim of this study was to assess stress-strain index (SSI) and corneal biomechanical parameters in eyes with granular corneal dystrophy (GCD). Methods: This case-control study included 12 eyes of 12 patients with GCD (mean age 45.2 ± 18.7 years) and 20 eyes of 20 healthy individuals (mean age 54.4 ± 3.8 years). In addition to SSI, dynamic corneal response (DCR) parameters were assessed at the first and second applanation, including length (AL1, AL2), velocity (AV1, AV2), time (AT1, AT2), and deformation amplitude (DA A1, DA A2), and at the highest concavity (HC) phase, including DA, peak distance (PD), radius (HCR), and DA ratio (DAR 1 and 2 mm), by Corvis ST. Central corneal thickness (CCT) and biomechanically corrected intraocular pressure (bIOP) were considered covariates in comparing DCR parameters between the two groups. Results: SSI was statistically significantly lower in eyes with GCD than in normal eyes (*p* = 0.04). The corneal velocity towards the first applanation was 0.02 m/s faster in the GCD eyes AV1 (0.15 ± 0.02 vs. 0.13 ± 0.02 m/s, *p* < 0.001) and IR (7.48 ± 1.01 vs. 6.80 ± 1.22 mm, *p* = 0.003) parameters were significantly higher in the GDC group, while AT1 (7.33 ± 0.66 vs. 7.47 ± 0.36 ms, *p* = 0.002) and HCR (7.42 ± 0.76 vs. 8.20 ± 1.08 mm, *p* = 0.014) were significantly lower in the normal group. Conclusions: GCD led to a change in biomechanical properties of the cornea. SSI refers to fewer stiff corneas in GDC than normal.

## 1. Introduction

Granular corneal dystrophy (GCD) is a stromal dystrophy with an autosomal dominant inheritance pattern [1]. Its onset is in the first decade of life and regardless of its type, it was reported as a common dystrophy in clinical practice [2,3,4,5]. GCD is usually characterized by central anterior stromal snowflake- or breadcrumb-like deposits, which progress slowly by increasing in the number of deposits and their peripheral spreading while the limbus remains uninvolved [2,3,4,5]. Consequently, due to the reduced corneal transparency secondary to opacification, the corrected distance visual acuity (CDVA) is diminished [6]. Recent studies showed that these opacities migrate from the epithelium to the inner layers of the stroma; however, GCD is classified as a stromal dystrophy [7,8].

Studies reported changes in the corneal biomechanical parameters in different types of corneal dystrophies [9,10,11]. Kamiya et al. mentioned no difference in the biomechanical parameters evaluated using an ocular response analyzer (ORA) between GCD and normal eyes [9]. As we know, there are two instruments available for in vivo clinical assessment of corneal biomechanics: ORA (Reichert Ophthalmic Instruments, Buffalo, NY, USA) and Corvis ST (OCULUS Optikgeräte GmbH; Wetzlar, Germany) [12]. Due to some limitations of ORA, including the limited corneal area assessed and variable air pressure dependent based on the first applanation pressure (P1) applied, Corvis ST produces acceptable measurements with adequate reliability in an 8 mm area along the horizontal meridian [13,14].

Phototherapeutic keratectomy (PTK) and penetrating keratoplasty (PKP) are the most recommended visual rehabilitation methods for GCD cases with significant corneal opacities [15,16,17,18]. In addition to the histopathological changes in GCD, these surgical interventions may affect corneal biomechanical strength [19,20] and increase the rate of ectasia reoccurrence after surgery [21,22], so the assessment of corneal biomechanics in this group of disorders seems reasonable. Therefore, the current study was designed to compare corneal biomechanical parameters assessed using the Scheimpflug technology in GCD patients with healthy subjects.

## 2. Materials and Methods

This retrospective cross-sectional study included 12 eyes of 12 GCD patients and 20 eyes of 20 normal subjects. The sample size was calculated based on the mean and standard deviation of corneal biomechanical parameters obtained from a pilot study including 10 eyes in each group, considering a 95% confidence interval and 80% statistical power.

**Figure 1:**
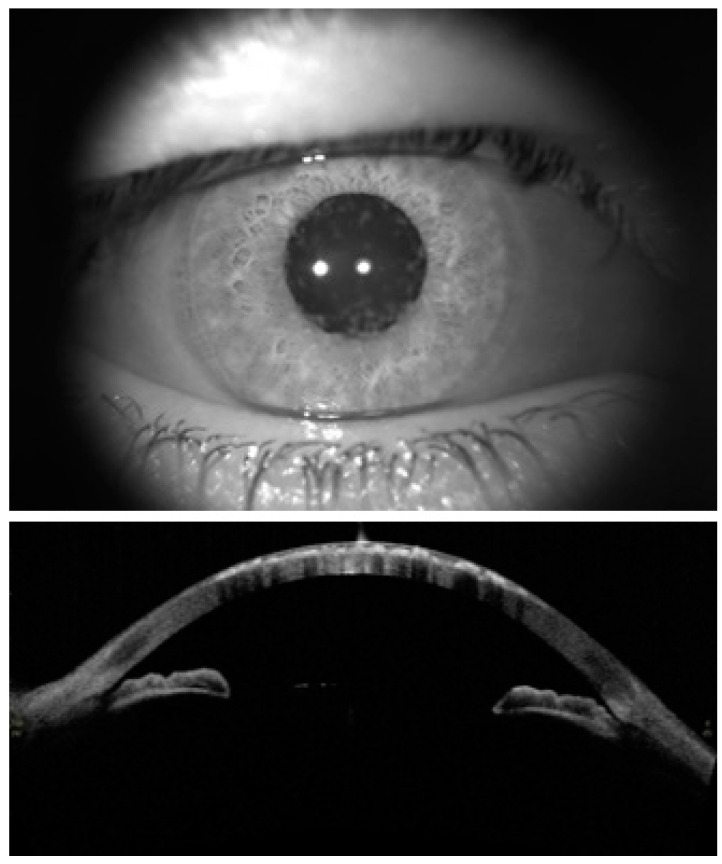
Slit-lamp biomicroscopy (diffuse illumination) (upper image) and anterior segment OCT (AS-OCT) images recorded from a case with GCD in the current study (lower image).

GCD was diagnosed based on the clinical finding of the presence of round whitish deposits in the anterior stroma in slit-lamp biomicroscopy using low-level light intensity in the retro-illumination technique, which was confirmed by an experienced corneal specialist (K.J.) (Figure 1). Although the accurate diagnosis of various types of GCD is almost impossible without appropriate genetic and histopathological tests [2,3], in the present study, clinical diagnosis was also aided by anterior segment optical coherence tomography (AS-OCT, Tomey CASIA SS-1000, Tomey Corporation, Nagoya, Japan). All steps of this study followed the tenets of the Declaration of Helsinki and a local ethics committee approved the study protocol (Code No.: IR.SEMUMS.REC.1401.043).

The inclusion criterion in the study group was clinically confirmed GCD. The control group was selected from the normal eye subjects who had visited the Vision Health Research Center, Tehran, Iran. In both groups, subjects with a corneal scar, moderate or severe dry eye, pterygium, chalazion, smoking history, pregnancy at the time of examination, history of corneal or ocular surgery, systemic or topical medications, contact lenses worn during the 2 weeks (for soft) or 4 weeks (for gas permeable lenses) before assessment, or history of any ocular or systemic disorders affecting the cornea, such as diabetes mellitus and collagen vascular disorders, were excluded from the study.

Along with a comprehensive ophthalmic examination, including visual acuity measurement, cycloplegic refraction, and anterior and posterior segment evaluations, corneal biomechanical parameters were assessed by Corvis ST for all subjects by a qualified and experienced technician during a single visit between 16:00 and 20:00 to avoid any bias.

The variables extracted from the Corvis ST printout are shown in Table 1 [23].

In addition to the above variables, a composite Corvis ST screening parameter known as the Corvis biomechanical index (CBI) was recorded. This index combines dynamic corneal response parameters with the pachymetric profile of the cornea along the horizontal meridian.

**Figure 2:**
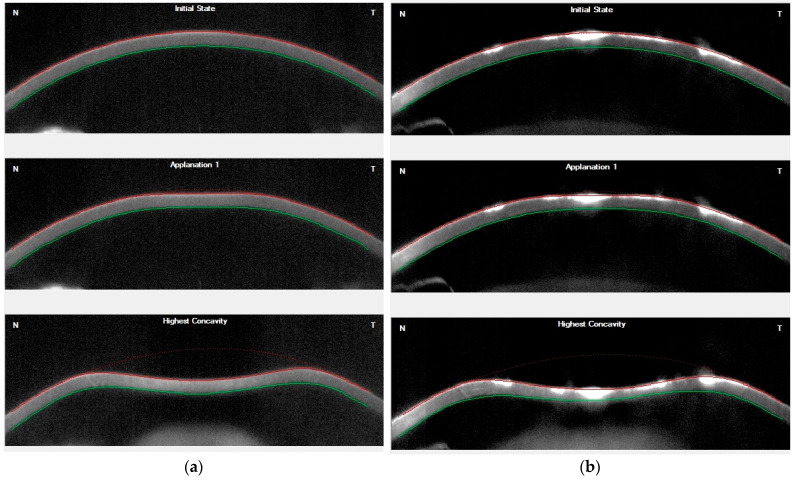
Corvis tomograms in (**a**) a normal subject; (**b**) a subject with granular corneal dystrophy (GCD).

Example of Corvis tomograms recorded in a normal case in the control group and a subject with GCD are shown in Figure 2.

Data were analyzed using SPSS software version 17. The normality of quantitative variables was checked by the Kolmogorov–Smirnov test. An independent sample *t*-test was used to compare age, CCT, and bIOP between the two groups. A general linear model was used to compare corneal biomechanical parameters in the normal and GCD groups, while CCT, bIOP, and age were considered covariates. Pairwise comparisons were performed using the Dunn–Bonferroni post hoc test. A *p*-value less than 0.05 was considered significant statistically.

## 3. Results

Forty-five percent of participants in the GCD group and fifty percent in the control group were males, with no significant difference in sex distribution between the two groups (*p* = 0.751).

The mean age was 43.6 ± 19.4 years (range: 14–75 years) in the GCD group and 45.7 ± 15.9 years (range: 20–63 years) in the control group, with no statistically significant difference between the normal and GCD subjects (*p* = 0.710).

The mean and standard deviation of CCT, IOPnct, and bIOP in GCD and normal eyes were 561.45 ± 68.12 and 542.65 ± 36.45 µm (*p* = 0.291), 18.62 ± 5.41 and 16.17 ± 2.90 mm Hg (*p* = 0.085), and 17.33 ± 5.69 and 15.53 ± 2.57 mm Hg (*p* = 0.210), respectively. 

The mean and standard deviation of standard and newer corneal biomechanical parameters of Corvis ST in the two groups are shown in Table 2 while CCT, bIOP, and age were considered covariates (Table 2).

Among all assessed biomechanical parameters, only AV1 (*p* < 0.001), AT1 (*p* = 0.002), HCR (*p* = 0.014), IR (*p* = 0.003), and SSI (*p* = 0.04) showed a statistically significant difference between the two groups using the independent samples *t*-test. The mean difference (GCD–Normal) was 0.02 m/s for AV1, −0.14 ms for AT1, −0.78 mm for HCR, 0.68 mm^−1^ for IR, and 0.11 for SSI.

Comparing the Ambrósio relational thickness to the horizontal profile (ARTh) in the two groups showed a statistically significant difference (*p* < 0.001), with mean values of 426.13 ± 206.23 microns (95% CI: 344.95 to 507.31) and 651.63 ± 168.90 microns (95% CI: 572.63 to 730.62) in GCD and normal eyes, respectively.

## 4. Discussion

To the best of our knowledge, few studies evaluated the corneal biomechanical properties using the Scheimpflug technology in granular corneal dystrophy (GCD). The main distinguishing feature of this study is the comparison of the newly introduced parameter of the stress-strain index in corneas with granular dystrophy with normal ones. Given that the biomechanical characteristics of the cornea as well as the dynamic corneal response of Corvis ST are affected by central corneal thickness, intraocular pressure, and age [12,24,25,26,27], the effect of these parameters in the comparison of corneal biomechanical parameters between these groups was controlled by considering them as covariates. The two groups were also homogeneous in terms of gender and age. The present study showed that, among all the assessed biomechanical parameters, only AV1 and AT1 (related to the first applanation phase), HCR and IR (related to the highest concavity phase), and SSI showed a statistically significant difference between the GCD and normal eyes. The corneal velocity towards the first applanation was 0.02 m/s faster in the GCD eyes and the first applanation phase was created 0.14 ms earlier than the normal eyes. These two findings point to the more elastic nature of the cornea, which has been reported in cases of keratoconus or corneal ectasia [28]. The lower corneal radius of curvature at the highest concavity phase also confirmed lower corneal strength and less rigidity of the cornea in the GCD group. The literature also shows that, in cases of corneal ectasia and with increasing severity, the integrated inverse radius (IR) increases [28,29]; this finding in GCD eyes agrees with previous reports. IR in the study group was 0.68 mm^−1^ higher than in normal eyes.

The stress-strain index (SSI) was introduced as a newer in vivo parameter to predict the biomechanical behavior of the cornea in terms of the material properties used in its structure [30]. Based on previous studies, the stress-stain behavior of biological tissue, such as the cornea, is nonlinear [31,32]. While biomechanical characteristics and corneal deformation response are affected by IOP and CCT [33,34,35], SSI was reported to be independent of these two parameters and only shows a significant positive correlation with age [30]. Moreover, it was mentioned as a better predictor of corneal stiffness, and values greater than or less than 1.0 indicate a biomechanically stiffer or softer cornea, respectively [36].

CBI was reported as a screening index to detect early ectasia even before the appearance of geometric changes in the cornea and higher values indicate a more likely presence of ectasia and less corneal stiffness [37,38]. The present study was designed on corneal biomechanical changes in a type of corneal dystrophy; however, the results show that GCD can cause an abnormal response in corneal biomechanical behavior compared to healthy corneas. This finding supports the higher CBI reported in cases with GCD compared to controls [39,40].

Histological evaluation showed that corneal collagen fibrils are maximally compressed in Bowman’s membrane and this layer plays a major role in corneal stability [41]. Therefore, it is expected that the biomechanical strength of the cornea in GCD is reduced secondary to the breaks in this layer due to the histopathological nature of the disease [19,20]. A lower SSI in GCD compared to normal corneas confirmed these changes in involved corneas. In addition, the present findings are in line with other studies that simultaneously reported the association of GCD with keratoconus [42,43,44,45,46].

Based on these findings and the presence of biomechanical instability of the corneas in GCD compared to normal corneas, including biomechanical evaluation in preoperative investigations is clinically valuable for deciding on the type and method of surgery in these patients to reduce the risk of iatrogenic ectasia following surgical interventions [21,22].

One study focusing on corneal biomechanical parameters in GCD was that of Kamiya et al., who used ORA to evaluate the biomechanical characteristics of GCD eyes before and after phototherapeutic keratectomy [9]. It was reported that the obtained ORA parameters (10.2 ± 2.2 mmHg for CH, 10.3 ± 2.0 mmHg for CRF) were similar to those of normal eyes (10.2 ± 1.3 mmHg for CH, 10.2 ± 1.7 mmHg for CRF) with equal CCT [9]. Considering the previously stated major drawbacks of ORA in evaluating corneal biomechanics, more studies are needed to confirm their findings.

A recent study that compared DCR parameters measured using Corvis ST in GCD and normal eyes reported higher A1DA and lower SPA1 in the GCD group [40], while these parameters were not statistically significant in the present study. In addition to the age difference, this difference can be attributed to racial differences (Chinese versus Iranian). Although their work points to abnormal corneal biomechanical behavior in GCD, unlike the present study, they did not evaluate the newly developed SSI parameter, which has been reported to be a better predictor of structural corneal strength.

In the present study, there was no significant difference in mean CCT between the two groups, while ARTh was significantly lower in the study group than the normal group, indicating an abnormal thickness profile or pachymetric progression in GCD patients, leading to a biomechanically softer cornea [47].

One limitation of the present study was the small sample size. A further study with greater numbers of patients is required to confirm our preliminary findings. Another challenge in our study is that some may argue for other covariates, such as the extent of the pathology, the number of deposits, or the area of the affected cornea, i.e., central, peripheral, or diffuse; however, the current sample size is too small to perform such classification and analysis. A study design considering the effect of these variables is suggested for future research.

## 5. Conclusions

GCD appears to alter corneal biomechanical characteristics assessed using Scheimpflug technology by Corvis ST. The corneal velocity towards the first applanation and the time taken to reach this phase, the corneal radius of curvature at the highest concavity phase, and the stress-strain index indicate less rigidity of the cornea in the GCD group. Until the availability of new biomechanical evaluation devices, such as the Brillouin microscopy in clinical practice, which can examine the biomechanics separately in each corneal layer, further studies are needed to confirm the present findings and the changes in the corneal biomechanical characteristics following different keratoplasty techniques in eyes with corneal granular dystrophy.

## Figures and Tables

**Table 1:** Parameters measured by Corvis ST included in this study.

Abbreviation (Unit)	Complete Name	Description of Specific Corvis ST Parameters
AL1 (mm)	Applanation length at the first flattening	Cord diameter of the flattened cornea at the first applanation
AL2 (mm)	Applanation length at the second flattening	Cord diameter of the flattened cornea at the second applanation
A1V (m/s)	First applanation velocity	Inward velocity of the cornea at the first applanation
A2V (m/s)	Second applanation velocity	Outward velocity of the cornea at the second applanation
A1T (ms)	Time at the first applanation	Time from initiation of air puff until the first applanation
A2T (ms)	Time at the second applanation	Time from initiation of air puff until the second applanation
A1DA (mm)	Deformation amplitude at the first applanation	Corneal displacement from the natural state until the first applanation
A2DA (mm)	Deformation amplitude at the second applanation	Corneal displacement at the second applanation
HC, PD (mm)	Highest concavity, Peak distance	Distance between the two peaks of the cornea at the highest concavity phase
HC, Radius (mm)	Highest concavity radius	Radius of curvature of the cornea at the highest concavity phase
HC-T (ms)	Highest concavity time	Time from initiation of air puff until highest concavity
HC, DA (mm)	Highest concavity, Deformation amplitude	Corneal displacement at the highest concavity phase
SPA1 (mm Hg/mm)	Stiffness parameter at the first applanation	Resultant pressure [adjusted pressure at A1 (adj AP1)-biomechanically compensated IOP (bIOP)] divided by deflection amplitude at A1
IR (mm^−1^)	Integrated inverse radius	Area under the inverse concave radius curve
DARMax1	Deformation amplitude ratio at 1 mm	Ratio between DA at the apex and the average of DAs at 1 mm around the center in temporal and nasal directions
DAR Max2	Deformation amplitude ratio at 2 mm	Ratio between DA at the apex and the average of DAs at 2 mm around the center in temporal and nasal directions
SSI	Stress-stain index	A parameter to predict the biomechanical behavior of the cornea in terms of the material properties used in its structure
CCT (µm)	Central corneal thickness	Corneal thickness at the corneal apex
IOPnct (mmHg)	Non-corrected IOP	Intraocular pressure non-corrected based on the corneal characteristic and age
bIOP (mmHg)	Biomechanically corrected intraocular pressure	Estimates IOP based on an algorithm that reduces the confounding effects of corneal characteristics and aging

**Table 2:** Mean and SD of the corneal biomechanical parameters using the Corvis ST separately in the two groups with the central corneal thickness (CCT) and biomechanically corrected intraocular pressure (bIOP), with age as covariates.

Variables	Mean ± SD (95% CI)		*p*-Value
	GCD (n = 12 Eyes)	Control (n = 20 Eyes)	
**AL1 (mm)**	2.55 ± 0.24 (2.45 to 2.65)	2.58 ± 0.21 (2.48 to 2.68)	0.704
**AL2 (mm)**	3.06 ± 0.49 (2.64 to 3.49)	3.46 ± 1.09 (3.05 to 3.87)	0.193
**AV1 (m/s)**	0.15 ± 0.02 (0.14 to 0.15)	0.13 ± 0.02 (0.12 to 0.13)	<0.001
**AV2 (m/s)**	−0.26 ± 0.06 (−0.28 to −0.25)	−0.27 ± 0.05 (−0.29 to −0.25)	0.872
**AT1 (ms)**	7.33 ± 0.66 (7.26 to 7.39)	7.47 ± 0.36 (7.41 to 7.53)	0.002
**AT2 (ms)**	21.02 ± 0.61 (20.89 to 21.14)	21.08 ± 0.29 (20.96 to 21.21)	0.471
**A1DA (mm)**	0.15 ± 0.02 (0.14 to 0.15)	0.14 ± 0.01 (0.14 to 0.15)	0.108
**A2DA (mm)**	0.43 ± 0.05 (0.40 to 0.46)	0.40 ± 0.07 (0.37 to 0.43)	0.144
**HC, PD (mm)**	4.99 ± 0.45 (4.90 to 5.08)	4.99 ± 0.27 (4.90 to 5.07)	0.955
**HC, R (mm)**	7.42 ± 0.76 (6.99 to 7.85)	8.20 ± 1.08 (7.79 to 8.62)	0.014
**HC-T (ms)**	16.59 ± 0.51 (16.38 to 16.80)	16.60 ± 0.35 (16.39 to 16.81)	0.948
**HC, DA (mm)**	1.05 ± 0.16 (1.01 to 1.09)	1.00 ± 0.11 (0.97 to 1.04)	0.123
**SP-A1 (mm Hg/mm)**	104.03 ± 19.29 (99.35 to 108.72)	110.45 ± 17.13 (105.90 to 115.01)	0.061
**IR (mm^−1^)**	7.48 ± 1.01 (7.18 to 7.78)	6.80 ± 1.22 (6.51 to 7.09)	0.003
**DAR Max2**	3.77 ± 0.42 (3.66 to 3.89)	3.65 ± 0.49 (3.54 to 3.76)	0.154
**DAR Max1**	1.51 ± 0.06 (1.49 to 1.53)	1.50 ± 0.06 (1.48 to 1.51)	0.259
**SSI**	1.09 ± 0.20 (1.00 to 1.17)	1.21 ± 0.17 (1.13 to 1.30)	0.04
**CBI**	0.33 ± 0.03 (0.31 to 0.35)	0.02 ± 0.03 (0.01 to 0.03)	<0.001

SD: Standard deviation; CI: confidence interval; AL: applanation length; AV: applanation velocity; AT: applanation time; DA: deformation amplitude; PD: peak distance; HC: highest concavity; T: time; R: radius of curvature, SPA 1: stiffness parameter at the first applanation; IR: integrated inverse radius; DAR: deformation amplitude ratio; SSI: stress-stain index; CBI: Corvis biomechanical index.

## Data Availability

All data generated or analyzed during this study are included in this published article.
